# Public health leadership: Competencies to guide practice

**DOI:** 10.1177/08404704211032710

**Published:** 2021-10-03

**Authors:** Tina Strudsholm, Ardene Robinson Vollman

**Affiliations:** 1 School of Health Sciences, University of Northern British Columbia, Prince George, British Columbia, Canada.; 2 Department of Community Health Sciences, Cumming School of Medicine, University of Calgary, Calgary, Alberta, Canada.

## Abstract

In 2013, the Community Health Nurses of Canada in partnership with the Canadian Institute of Public Health Inspectors and the Manitoba Public Health Managers Network received funding from the Public Health Agency of Canada to develop a set of interdisciplinary leadership competencies for seven public health disciplines. The Leadership Competencies for Public Health Practice in Canada project comprised a multimethod research approach that included a scoping literature review, on-line survey, webinar-based focus groups, and a modified Delphi process. The 49 leadership competencies for public health practice were organized according to the LEADS Canada capabilities. The leadership competencies extend the core public health competencies and discipline-specific competencies and reflect foundational values of public health. The leadership competencies can be applied to professional development pathways, mentoring programs, and performance appraisals to advance public health practice. How these competencies have been enacted by public health leaders during the COVID-19 pandemic is discussed.

## Introduction

Beginning in early 2020, Canada’s federal, provincial and territorial Chief Medical Officers of Health have been in the news almost daily, providing updates on the COVID-19 pandemic. The increased visibility of our public health leaders and attention to their role in the pandemic response have prompted reflection on what leadership means in public health practice in Canada. A thorough investigation of how public health leadership is demonstrated in Canada was initiated in 2013. The Community Health Nurses of Canada, in partnership with the Canadian Institute of Public Health Inspectors and the Manitoba Public Health Managers Network, received funding from the Public Health Agency of Canada for a three-year project to develop a set of interdisciplinary leadership competencies—essential knowledge, skills, and attitudes necessary for seven public health disciplines: dentistry, epidemiology, health promotion, inspection, medicine, nursing, and nutrition.

The Leadership Competencies for Public Health Practice in Canada (LCPHPC) project comprised a scoping literature review, on-line survey, and webinar-based focus group discussions to generate a comprehensive list of competencies, followed by a modified Delphi process to refine and organize the list prior to publication.^1^ Guiding the project team were a Project Steering Committee of the partners and an Expert Advisory Committee representing the seven public health disciplines and key associations representing public health in Canada.

In this article, we present a review of leadership literature and describe the methods employed to characterize the LCPHPC. We also discuss examples and potential practical applications of these emergent competencies.

A review of the leadership literature related to healthcare and public health practice in Canada revealed an area of research replete with countless attributes, skills, and knowledge of ideal leaders but also an absence of shared definitions, theoretical foundations, leadership frameworks, or empirical study on the impact of ideal leadership competencies. Furthermore, the terms *leadership* and *management* are regularly conflated in the literature, yet it is clear in public health practice that leadership is not management. Leadership in public health can be observed, demonstrated, and fostered at all levels of an organization, irrespective of power and formal responsibilities afforded to management positions within a bureaucracy. From this nebulous field of research, a wayfinding characterization of effective leadership has emerged.^2^ This framework comprises five general categories of leadership competencies (also termed capabilities) related to managing self, building teams, achieving results, creating coalitions, and transforming systems.^3^

Beyond the five categories of leadership competencies, there are personal characteristics that benefit leaders. In light of a public health practitioner’s mandate to work across multiple sectors, often beyond that of healthcare, and the necessity to navigate diverse and at times divergent views and priorities, affable leaders enjoy a great advantage. Effective leaders, while often working to the scope of their role and beyond, readily acknowledge and lean on the expertise and knowledge of others as appropriate.^4^ Public health leaders act with empathy^5^ but also with the greater good in mind. Although leaders must acknowledge organizational needs and priorities, ultimately the needs of the population usurp the needs of the organization and must be kept at the forefront of decisions and actions.^6^

There is limited literature exploring the impact of individuals demonstrating ideal public health leadership competencies, or how the environments within which public health leaders work promote or impede leadership. However, public health crises, such as the SARS (severe acute respiratory syndrome) outbreak in 2003 and the current COVID-19 pandemic, offer natural experiments from which to glean insights about the effectiveness of public health leadership, and what environments support or hamper leaders. During SARS, Naylor and colleagues^7^ identified the need and utility of public health leadership:

SARS has highlighted how communicable diseases, [in] particular those caused by hitherto unknown agents, can tap primal anxieties, prompt enormous interest on the part of the media, and provoke some unsavoury public responses (e.g., incidents of harassment and scapegoating of the Asian community in Toronto). The SARS outbreak thereby underscores the need for public health to play a leadership role in analyzing risks and communicating effectively about them. (p. 64)

Despite the observed importance and potential of leadership in public health, the concept has not been defined clearly. The LCPHC project aimed to identify the specific knowledge, skills, and abilities of public health leaders so as to operationalize leadership in public health practice in Canada. The following presentation of the multimethod approach to the project describes a robust identification of leadership competencies based on available evidence and extensive consultation with public health professionals across seven public health disciplines. The discussion describes opportunities for practical application of the competencies and select examples of LCPHPC evidenced during the COVID-19 pandemic.

## Methods

The LCPHPC project was composed of four distinct parts—scoping literature review, on-line survey, webinar-based focus group discussions, and a modified Delphi process, each approved by the Conjoint Health and Ethics Review Board (CHREB) at the University of Calgary. Each part built upon the results of the preceding parts to develop a comprehensive list of leadership competency statements supported by Canadian public health professionals from seven disciplines.

### Scoping literature review

A scoping review^8^ of both scholarly and grey literature was undertaken to identify evidence and theories relevant to public health leadership competencies, including enablers and barriers to practising leadership in public health.^9^ Scholarly literature was retrieved through customized keyword searches of electronic databases. Supplementary literature was identified in consultation with experienced public health professionals. Results were limited to English publications from 1995 to 2013; 3298 citations were retrieved. Of these, after screening for relevance, 139 full-text articles were retained. Publications were from more than 25 different countries, with most being from the United States, the United Kingdom, and Canada. Public health nursing was most often represented in the literature (41%).

Review of the literature identified desirable leadership qualities (personal traits, knowledge, skills, behaviour), leadership tasks and activities, as well as enablers and barriers of leadership (personal, organizational, community, and system levels). Ultimately, the result of the scoping review was the identification of 80 leadership competencies for public health practice.

### On-line survey

An on-line survey validated the results of the literature review. The Expert Advisory Committee members who represented the professional associations of the public health disciplines included in the LCPHPC project distributed the survey link via e-mail to the memberships of their respective professional associations.^10^ The survey link included a formal invitation to participate and details to obtain written informed consent to meet CHREB requirements. Using a modified Dillman Total Design Survey Method,^11^ reminders to distribute the survey link were sent three times. The survey was open for 8 weeks.

There were 821 responses to the on-line survey, with a completion rate of 72%. The overall response rate was 18%. Response rates varied across disciplines; the average response rate was 26%, ranging from a high of 39% in community/public health nursing to a low of 8% in environmental health/inspection. Most respondents were frontline staff members (42%) or individuals in first-level management positions (11%).

The survey presented leadership characteristics identified in the scoping review^9^ in three lists: knowledge, skills, and behaviours. Respondents were asked to identify the top 5 characteristics within each list that described “good leaders” in public health. Respondents were asked to identify the top 5 barriers and enablers to public health leadership.^9^ Feedback in the open-text comment sections validated that the descriptors in the survey were comprehensive; although some people used different terminology, it was synonymous with the words found in the literature and listed in the survey. In terms of prioritizing, respondents clearly ranked the top five in each list, including enablers and barriers to exercising leadership.

### Webinar-based focus group discussions with public health leaders

The results of the on-line survey^10^ provided the basis for planning the focus group process.^12^ Focus group participants were nominated by the Expert Advisory Committee as leaders in public health from across Canada in their respective disciplines. From the list of 92 nominees, 27 purposively selected participants took part, including from remote, rural, and northern settings, as well as from francophone and Indigenous groups. Of particular note was the large representation of mid- and senior-level managers nominated (60%) and participating (49%), compared to the predominance of frontline staff and first-level managers (53%) that took part in the on-line survey.

Five focus group webinars were conducted over a three-week period. Informed verbal consent was obtained to fulfill CHREB requirements. The top five statements from the survey^10^ (from each category) were presented for discussion. In general, the statements were accepted with only minor suggestions for clarity with participants also contributing additional dimensions of public health leadership practice.

### Delphi process

A modified Delphi process was used to determine the level of agreement and to obtain feedback on the draft set of competency statements distilled from the first three parts of the project.^13^ A panel of experts was recruited by the nomination of 10 leaders from each of the seven participating discipline associations for a total sample of 70 panelists, taking into consideration geographic location, age, sex, and years of experience in public health practice. Of the initial 70 panelists, 48 continued to the end of the Delphi process.

Three survey rounds were conducted, asking panelists to rate their level of agreement with the proposed competency statements (on a 5 point Likert scale) and to provide comments related to each individual statement. At the close of each round, all responses were collated and analyzed, and statements were either accepted or deleted from the survey by consensus or revised for the next round based on comments included in the survey. Consensus was defined as an expression of agreement (strongly agree or agree on the Likert scale) by at least 80% of the panelists, and those competency statements that received an 80% or higher level of agreement were accepted as final. At the completion of the three rounds of the modified Delphi process, 49 statements were accepted as LCPHPC (Table 1).

## Results

With the research phases of the LCPHPC project completed, the public health leadership competencies were organized for presentation according to the LEADS framework.^2^ This framework was chosen to be consistent with other sectors of the Canadian health system, fostering a common understanding of leadership and the ability to communicate across settings.

**Table 1.** Public health leadership competencies arranged according to the LEADS framework

Lead self: *Self-motivated leaders are self-aware, manage themselves, develop themselves, and demonstrate character*	Public health leaders: Abide by the ethical codes of their respective disciplines and also to the ethics relevant to public health practice Critically examine their role within the public health sector organization and within regulatory systems Demonstrate evidence-informed decision-making Demonstrate lifelong learning and self-development Are accountable Demonstrate emotional intelligence Are self-aware and reflective Demonstrate reflexivity and flexibility in response to criticism
Engage others: *Engaging leaders foster the development of others, contribute to the creation of healthy organizations, communicate effectively, and build teams*	Public health leaders: Leverage communication technologies, as appropriate, to communicate effectively (e.g., audio/videoconferencing, webinars, social media, e-mail, program-specific software, etc.) Demonstrate transdisciplinary understanding of the multiple professions with which they collaborate Are credible Tailor their communication to respect different audiences Engender respect, rapport, and trust Empower and enable others by providing strong, unwavering support Are responsive and accessible Build capacity through modelling and mentorship for leadership in others Promote a healthy workplace culture Share power horizontally and vertically Apply a variety of decision-making styles appropriate to the context Build consensus where appropriate Mobilize others Possess effective negotiation skills Possess effective mediation skills Recognize and encourage contributions of others Communicate clearly and transparently up and down and across the organizational hierarchy
Achieve results: *Goal-oriented leaders set direction, strategically align decisions with vision, values, and evidence, take action to implement decisions, and assess and evaluate*	Public health leaders: Use their understanding of power and influence and operational expertise to mobilize people and networks to meet strategic objectives Garner support for and momentum to a public health vision of upstream solutions to health issues Share a personal vision that is explicit, clear, and compelling Anticipate and take advantage of leadership opportunities Champion public health principles, actions, and interventions Assess program effectiveness and success in terms of population health (vs. business models)
Develop coalitions: *Collaborative leaders purposefully build partnerships and networks to create results, demonstrate a commitment to customers and service, mobilize knowledge, and navigate socio-political environments*	Public health leaders: Demonstrate cultural awareness of the implications of politics, ethnicity, gender, age, socioeconomic status, and religion on health beliefs and behaviours Demonstrate ability to guide healthy public policy decisions and processes Recognize the role of public health in political influence Are ambassadors of quality evidence-informed public health practice Foster engagement with communities Serve as catalysts to build partnerships, coalitions, increased capacity, and shared leadership Promote awareness and visibility of public health practice Contribute to cross-disciplinary understanding of the contribution of public health practice Leverage partnerships to broaden the scope and impact of public health practice (i.e., individual immunizations vs. population-based interventions
Systems transformation: *Successful leaders demonstrate systems/critical thinking, encourage and support innovation, orient themselves strategically to the future, and champion and orchestrate change*	Public health leaders: Demonstrate understanding of knowledge translation Demonstrate understanding of how to guide change Demonstrate systems thinking skills Demonstrate critical thinking skills Demonstrate innovation and creativity Advocate for and guide change Demonstrate drive and motivation Demonstrate forward thinking Adapt to rapidly changing public health sector and health systems

## Discussion

The four research phases of the LCPHPC project revealed a very strong consensus about the competencies for public health leadership. The various public health disciplines have different foreground knowledge bases, but the results indicate that interdisciplinary knowledge is valuable and important. To provide leadership in public health, leaders must know about the field of public health itself: population and public health (determinants of health, health demographics, and outcomes); public health values; ethics of public health; and inequalities, inequities, and social justice. This knowledge is embraced in the core competencies for Public Health^14^ and also in the seven discipline-specific competencies. The leadership competencies complement and extend the core and discipline-specific competencies and, as a result, the foundational values inherent in public health practice form the context within which leadership competencies are demonstrated.

There is strong agreement between what the literature says leadership entails and what the research participants said were characteristic of good leaders in public health. At a minimum, the competencies that participants agreed with public health leaders must have include:

- good communication skills (clarity, transparency and accountability, interdisciplinary) to engender trust and rapport, build teams, and partnerships;
- excellent supporting, empowering, and capacity building skills with the ability to model and mentor leadership growth in others and build consensus;
- clear critical thinking skills that utilize evidence to inform decisions; and
- initiative, transparency, and forward thinking that drives a positive, ethical, and socially just public health agenda.

There is no question that in order to carry out the demands of building teams and partnerships a leader must be responsive and accessible. However, organizations frequently impose constraints to accessibility such as lack of staffing that requires leaders to carry out other tasks that reduce the time needed to foster relationships and lack of recognition of the dedicated time needed for the work of leadership. Adding to that, research participants reported a lack of mentoring and educational opportunities to develop public health leadership capacity. In addition, leadership is difficult when many staff are burned out or some are simply oppositional and do not support change. For public health leadership to flourish, leaders must have the ability to adapt to a changing system and to be innovative, creative, and flexible. Such adaptation requires critical thinking skills and evidence-informed decision-making that must go hand-in-hand with innovation. In this, participants emphasized that a level of political proficiency is an essential ability of public health leaders, particularly in today’s domination of the health system by the acute care sector.

Participants suggested that the Public Health Agency of Canada’s definition of leadership^14^ should include the desired outcomes of public health leadership: equity, social justice, and engagement. Further, they supported the need for a common framework for leadership competencies across all sectors of the health system to encourage a common language, facilitate communication, and bring people from across the health system together in learning opportunities, rather than separating them out by discipline, health sector (public health, long-term care, acute care), or work setting. Since leadership is exercised at all levels of the system, participants recommended leadership competencies be developed for novice, intermediate, and advanced stages of leadership, where more seasoned leaders can become mentors for novice leaders.

Implementation of these leadership competencies requires that the various public health disciplines embrace them in addition to the core and discipline-specific competencies and promulgate them among their memberships. The Community Health Nurses of Canada have used them as a framework for its annual conferences as a means of raising awareness. Its mentoring system has paired experienced leaders with members wishing to increase their leadership ability in a successful effort of leadership capacity development for community health nurses. We are aware that some public health employers are using the competencies in their various human resource and management processes (e.g., job descriptions, hiring, performance appraisals, and continuing education). Several educational programs are introducing the leadership competencies to their senior students, residents, and graduate students (e.g., Pubic Health and Preventive Medicine, Master in Public Health). Although the uptake of the leadership competencies is not universal in public health organizations in Canada, there are positive indications from the number of sales of the competency document that there is growing interest in leadership capacity development in the public health workforce.

To use the competencies effectively, there must be room to interrogate them to determine fit in the organizational, cultural, and environmental context where they are intended to be applied. For instance, they do not include a competency for critical social theory knowledge, long considered a foundation of public health practice. Is this an important capacity needed in your setting? Some competencies such as a sense of humour and calmness under pressure were suggested but did not make the competency list. Are these capacities part of the “characteristics” valued in leaders or competencies required to be a successful leader? Yet, being cool under pressure, composed and unruffled when challenged, and using humour judiciously have certainly been hallmarks of our public health leaders on television and social media during the COVID-19 pandemic. Canada’s Chief Public Health Officer and the provincial/territorial Medical Officers of Health have demonstrated the leadership competencies in action as they provided updates to the public and advice to political leaders during the COVID-19 pandemic.

## Conclusion

In times of sweeping uncertainty like the COVID-19 pandemic, leadership from public health practitioners becomes indispensable.^15,16^ The public health leadership competencies represent a set of skills and expertise necessary to tackle salient issues arising from the COVID-19 pandemic response including public trust, health literacy, inequitable burden of disease, data monitoring/access/integration/reporting, and cooperation with the global community.^17,18^ In fact, early reflections on the COVID-19 response identify the important contributions of specific leadership competencies in public health practice such as systems thinking and evidence-informed decision-making.^17,19^

Adoption of the competencies for public health practice in Canada is a stepping stone toward strengthening public health leadership and, ultimately, advancing public health goals such as improved health outcomes and health equity. Sustained empowerment of public health leaders will require additional attention to contexts that enable or hinder leadership capacity and impact. As public health practitioners aim to influence determinants of health by working across social structures and systems, research guided by critical social theory may illuminate how leadership competency implementation intersects with organizational, cultural, and environmental contexts. The responsible and recommended focus in pandemic recovery should be to address how to create resilient communities supported by globally empowered systems in which public health leadership can thrive.

### Limitations

The leadership competencies statements must not be seen as static but as dynamic representations that are subject to context, growth, and critique. For example, the statements are gender-neutral. But how are they expressed by different people in different situations? Do leadership styles vary across a gender spectrum? Does ethnic background influence understanding of leadership? How important is organizational culture to the ability to exercise leadership? The competencies might need updating over time or be curated according to the setting where they are implemented.
